# Supplementary material for: A Single-Nucleus Transcriptomic Atlas of the Mouse Lumbar Spinal Cord: Functional Implications of Non-Coding RNAs
Source: BioTech (Basel). 2025 Sep 3;14(3):70. doi: 10.3390/biotech14030070 (PMC12452356; doi:10.3390/biotech14030070)
Supplement: Supplementary file 1 [file biotech-14-00070-s001.zip › biotech-3823543-supplementary/biotech-3823543_SupplementaryMaterial/About_Suppl_Files.pdf]

Due to file size limitations, we were unable to upload the full supplementary material directly to the submission system; therefore, the supplementary files 1, 2, and 3 are provided via the following Google Drive link:

https://drive.google.com/drive/folders/1G1-p27Jrikt7TsxH16cyGQVm7ldxupo3?usp=drive_link

The supplementary material can also be found at OSF repository:

https://osf.io/xwfsz/
