# Supplementary material for: A Single-Nucleus Transcriptomic Atlas of the Mouse Lumbar Spinal Cord: Functional Implications of Non-Coding RNAs
Source: BioTech (Basel). 2025 Sep 3;14(3):70. doi: 10.3390/biotech14030070 (PMC12452356; doi:10.3390/biotech14030070)

Supplementary Figure 1: Clustree results of the three datasets (ALL, coding and non-coding) showing the clusters obtained at low resolutions (from 0.01 to 0.05). The red square indicates the chosen resolution for each dataset. The three resolutions lead to 11 clusters to ease comparisons.

Legends for the figures:

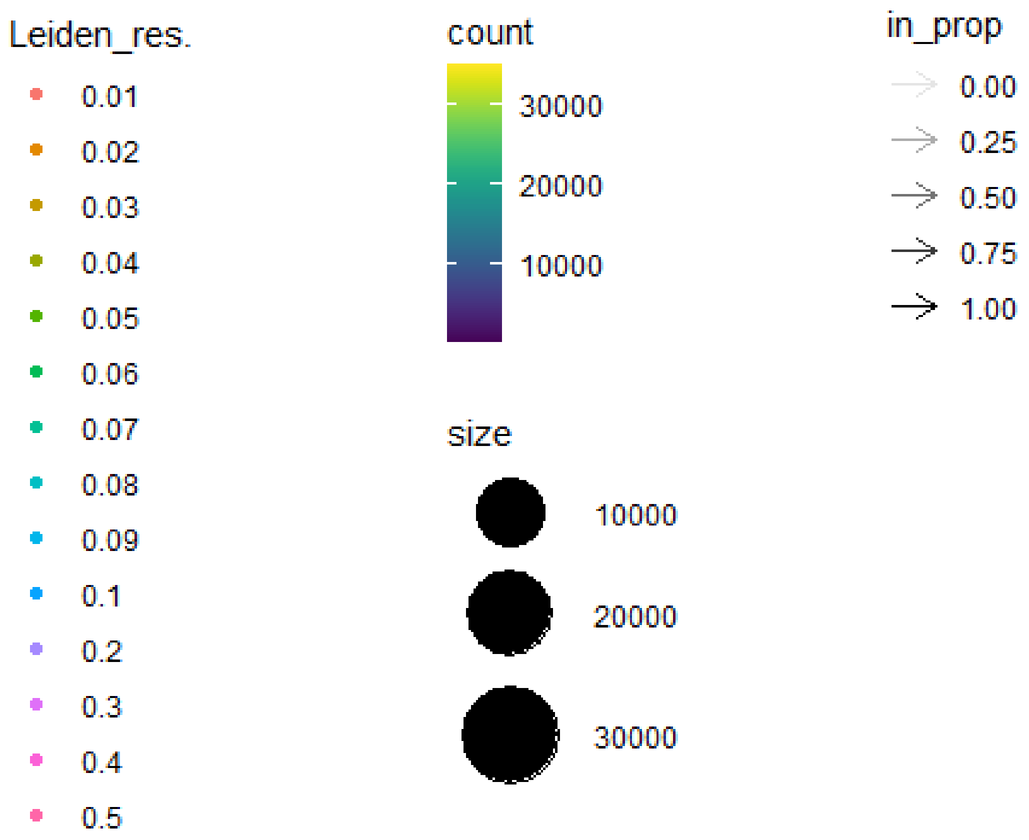

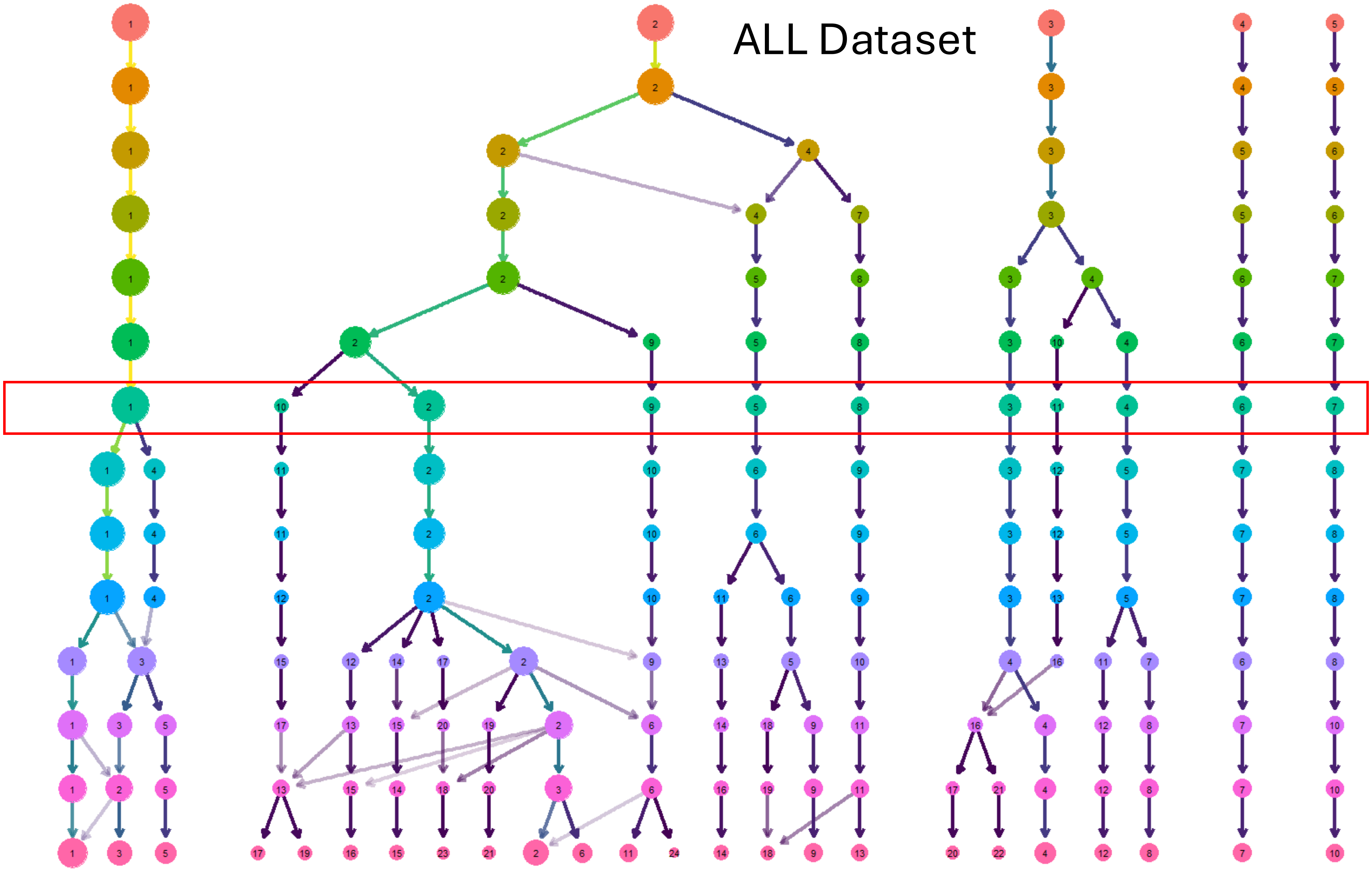
ALL Dataset

CG Dataset

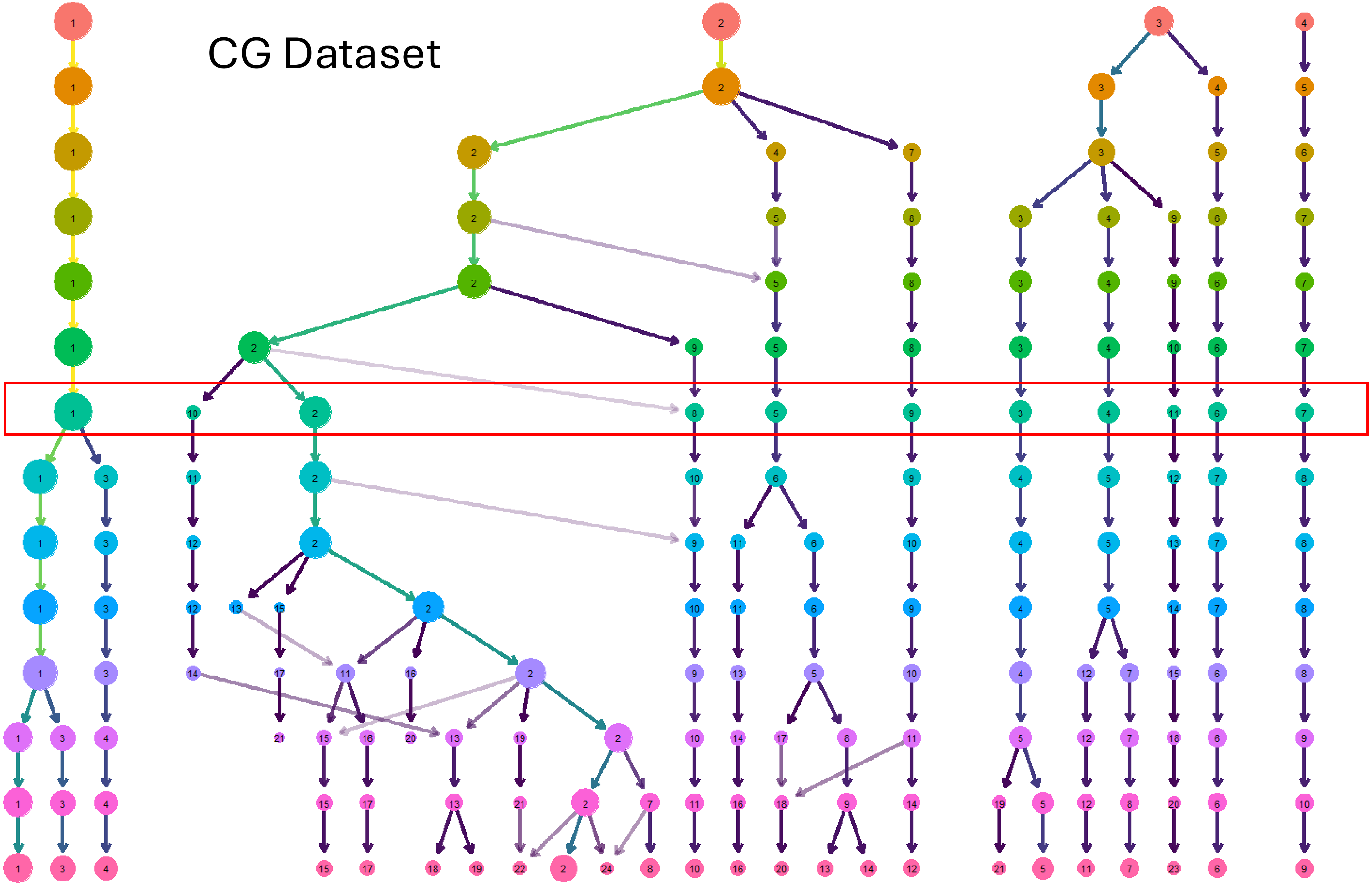

NCG Dataset

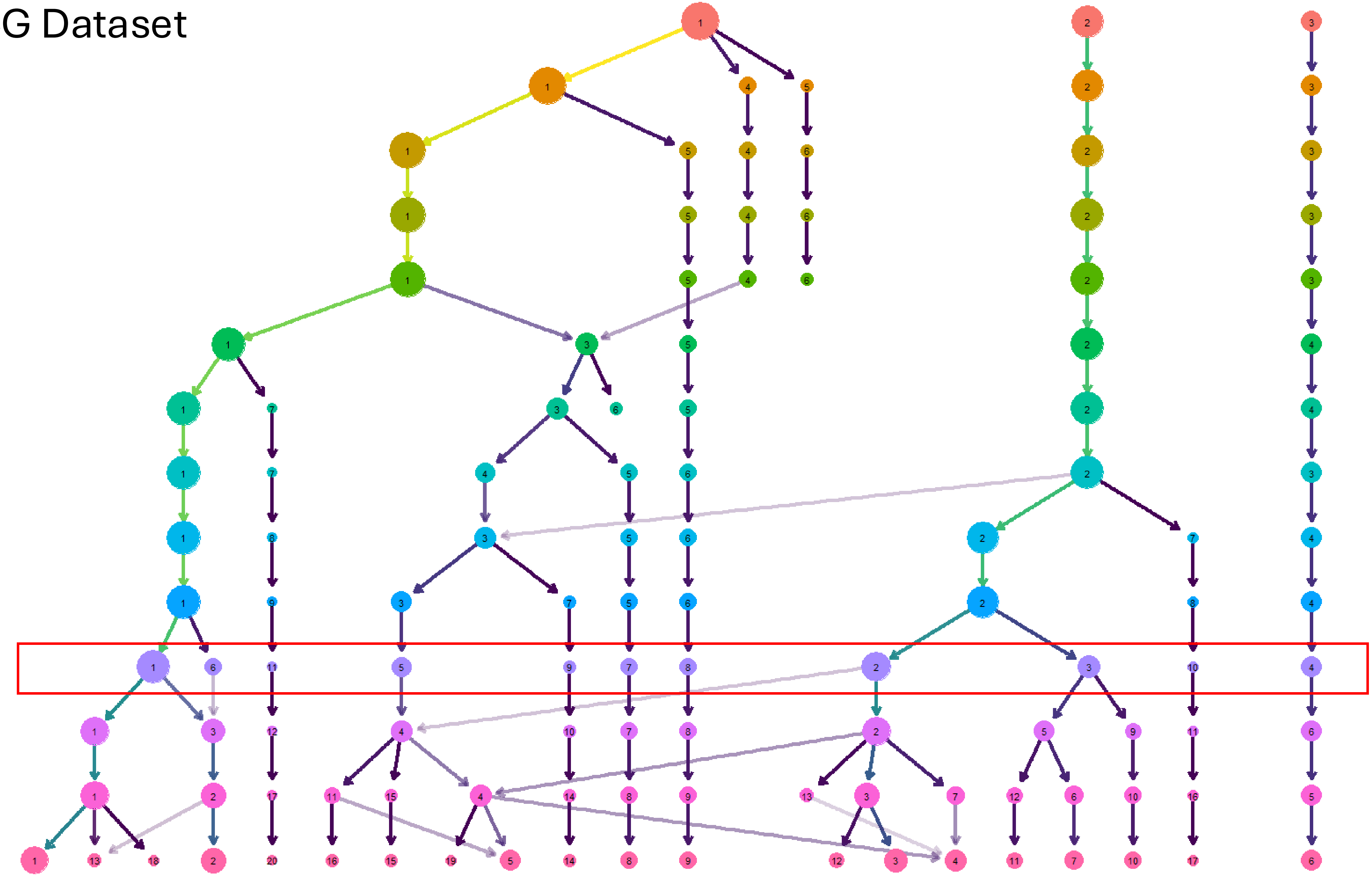

Supplement: Supplementary file 1 [file biotech-14-00070-s001.zip › biotech-3823543-supplementary/biotech-3823543_SupplementaryMaterial/New_Suppl_Fig_1.pdf]
