# Supplementary material for: A Single-Nucleus Transcriptomic Atlas of the Mouse Lumbar Spinal Cord: Functional Implications of Non-Coding RNAs
Source: BioTech (Basel). 2025 Sep 3;14(3):70. doi: 10.3390/biotech14030070 (PMC12452356; doi:10.3390/biotech14030070)

Supplementary Figure 2: Clustree results of the ALL dataset showing the clusters obtained at resolutions up to 1.5. The red square indicates the chosen resolution.

Legends for the figure:

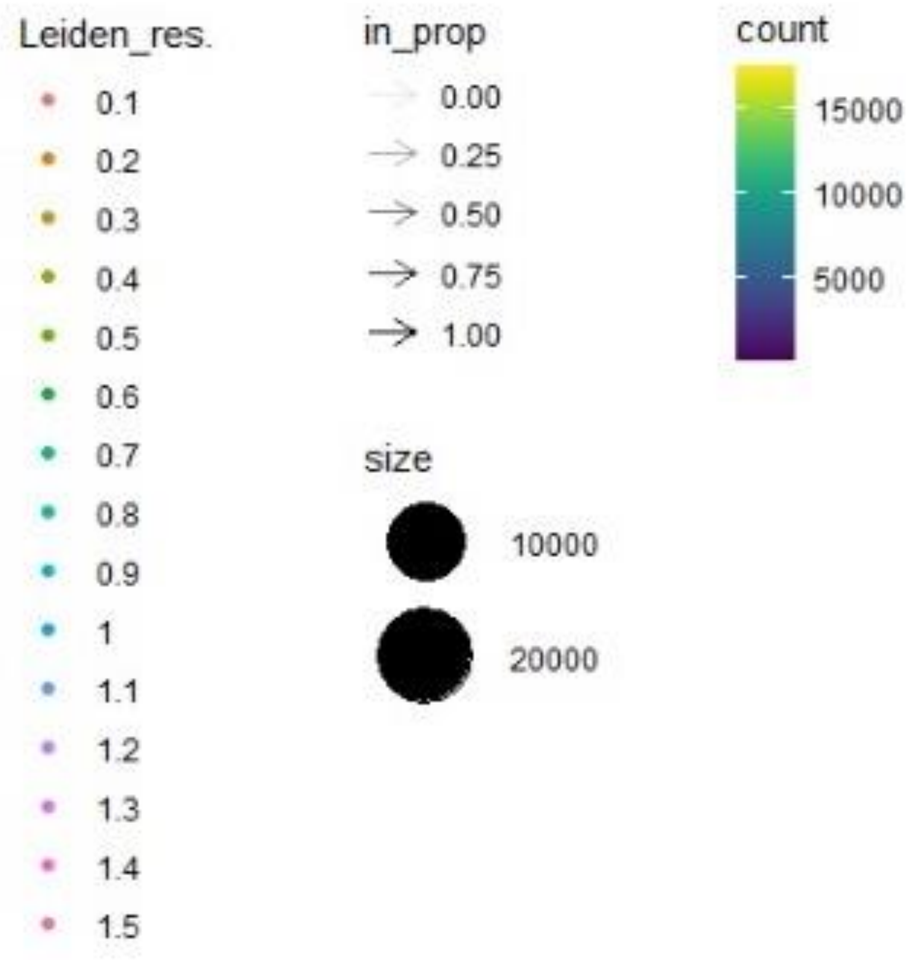

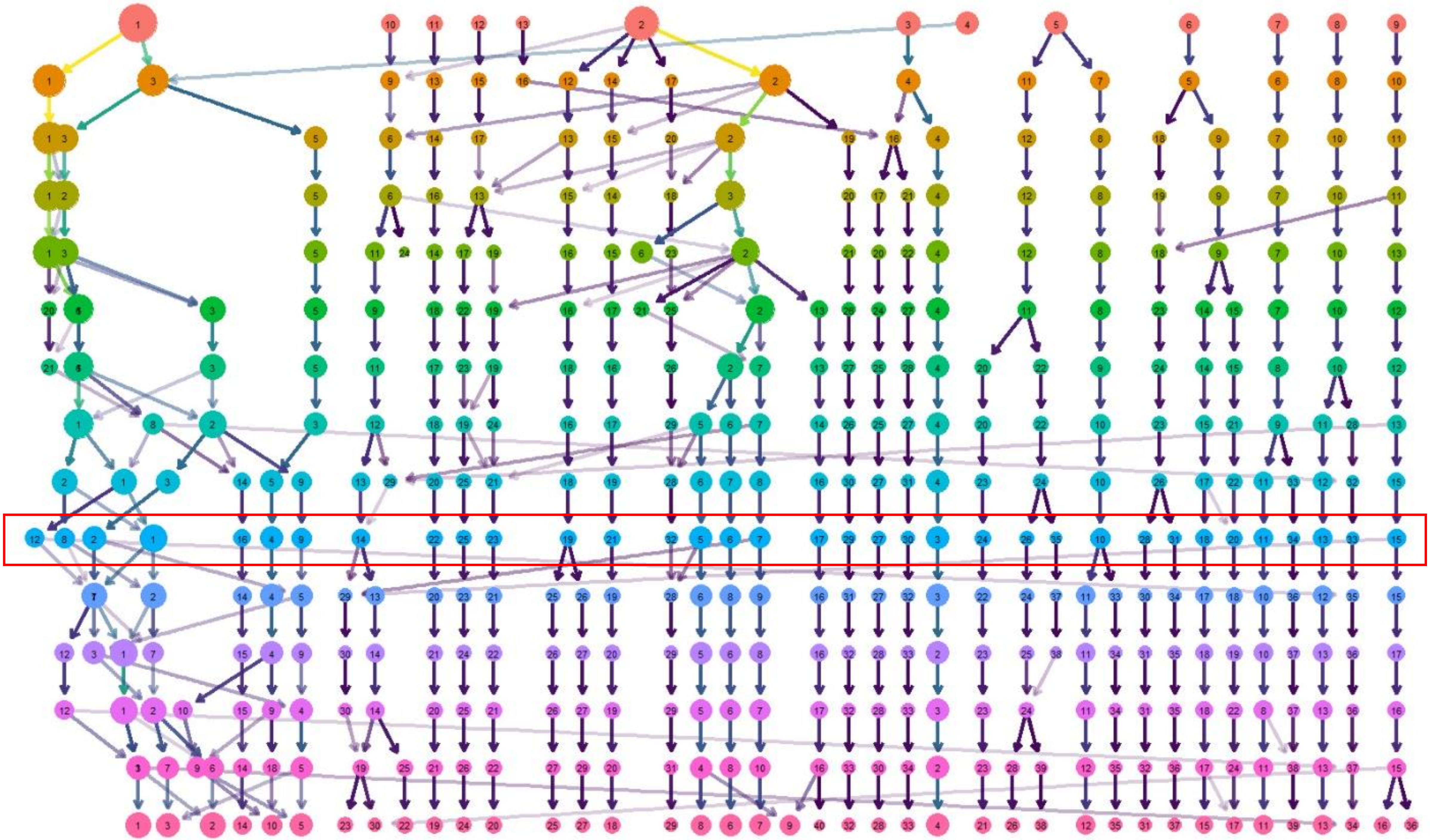

Supplement: Supplementary file 1 [file biotech-14-00070-s001.zip › biotech-3823543-supplementary/biotech-3823543_SupplementaryMaterial/New_Suppl_Fig_2.pdf]
