# Supplementary material for: A Single-Nucleus Transcriptomic Atlas of the Mouse Lumbar Spinal Cord: Functional Implications of Non-Coding RNAs
Source: BioTech (Basel). 2025 Sep 3;14(3):70. doi: 10.3390/biotech14030070 (PMC12452356; doi:10.3390/biotech14030070)
Supplement: Supplementary file 1 [file biotech-14-00070-s001.zip › biotech-3823543-supplementary/biotech-3823543_SupplementaryMaterial/New_Suppl_Table_4.pdf]

Supplementary Table 4. Clusters identified at high resolution.

Lineage	Cluster	Cells	Populations
Oligodendrocytes	Cluster_1	10,845	MOL, MFOL, NFOL
	Cluster_2	6,170	MOL, MFOL
	Cluster_4	5,652	MOL, MOL_ischemic
	Cluster_8	3,800	MOL, MFOL
	Cluster_9	3,552	MOL, MOL_ischemic
	Cluster_10	3,263	Mixed: Vascular_leptomeningeal_ECM_forming, OPC- differentiating endothelial_capillary, NSC
	Cluster_12	2,710	MFOL, NFOL
	Cluster_13	2,627	OPC, OPC_cycling
	Cluster_16	2,047	MOL, MFOL
	Cluster_33	322	COPs, NFOL
Astrocytes	Cluster_3	5,928	Astrocytes_protoplasmic/reactive
	Cluster_27	642	Astrocytes_fibrous
	Cluster_30	494	Ependymal
Immune cells	Cluster_11	3,102	Immune, Microglia, Macrophage, Neutrophil, Myeloid
	Cluster_34	305	B_cells, CD8_T_cells, Macrophage_border-associated, NK Neutrophils
Vascular cells	Cluster_24	1,312	Arachnoid, Leptomeningeal_ECM forming
	Cluster_26	892	Endothelial
	Cluster_35	203	Pericytes, Endothelial_venous
Neurons	Cluster_5	5,391	VENT, MED, DI_Npy_Vgf
	Cluster_6	4,433	VENT, MED, NSC
	Cluster_7	4,177	MEL, MEP, MN, VEL, VEP, VIP, DE_Rreb1_Zim1
	Cluster_14	2,587	DE_Rreb1_Zim1, DE_Sox5_Qrfpr, DE_Tac1
	Cluster_15	2,116	DE_Reln_Nmur2, DE_Rreb1_Zim1
	Cluster_17	1,971	Mixed neurons, CSF-cN
	Cluster_18	1,707	DE_Maf_Cpne4_Rorb, DE_Maf_Kcnh8
	Cluster_19	1,498	DI_Nxph1_Npy, DI_Rorb_Nppc, DI_Rorb_Rxfp2
	Cluster_20	1,496	DE_Nts, DE_Trh
	Cluster_21	1,476	DI_Maf_Adarb2, DI_Rorb_Adamts5
	Cluster_22	1,464	DE_Tac2_Nmu
	Cluster_23	1,365	DI_Maf_Adarb2, DI_Pdyn_Gal, DI_Rorb_Kcnip2, DI_Rorb_Rxfp2
	Cluster_25	995	DI_Gal
	Cluster_28	554	DE_Cck_Cpne4
	Cluster_29	534	VENT, MED, MN
	Cluster_31	424	DE_Reln_Trhr
	Cluster_32	324	DI_Npy_Qrfpr

Clusters are characterized by their number of cells, cell type and included populations. Cell population names according to *Tabulae Paralytica* nomenclature. VENT: Ventral neurons. MED: Medial neurons.
